# Modulation of *PTPN2/22* Function by Spermidine in CRISPR-Cas9-Edited T-Cells Associated with Crohn’s Disease and Rheumatoid Arthritis

**DOI:** 10.3390/ijms22168883

**Published:** 2021-08-18

**Authors:** Ameera M. Shaw, Ahmad Qasem, Saleh A. Naser

**Affiliations:** Division of Molecular Microbiology, Burnett School of Biomedical Sciences, College of Medicine, University of Central Florida, 4110 Libra Drive, Orlando, FL 32816, USA; amshaw@knights.ucf.edu (A.M.S.); ahmadqasem@knights.ucf.edu (A.Q.)

**Keywords:** Crohn’s Disease, Rheumatoid Arthritis, PTPN2, PTPN22, spermidine, polyamines

## Abstract

Crohn’s Disease (CD) and Rheumatoid Arthritis (RA) share some single nucleotide polymorphisms (SNPs) in protein tyrosine phosphatase non-receptor types 2 and 22 (*PTPN2/22*). Recently, we reported that clinical samples from CD and RA patients associated with *PTPN2:rs478582* or *PTPN22:rs2476601* genotypes were linked to overactive immune response and exacerbation of inflammation. Here, we investigated in vitro the effects of these SNPs in Jurkat T-cells using CRISPR-Cas9. All cells were evaluated for *PTPN22/22* loss of function and effects on cell response. We measured gene expression via RT-qPCR and cytokines by ELISA. We also measured cell proliferation using a BrdU labeling proliferation ELISA, and T-cell activation using CD-25 fluorescent immunostaining. In *PTPN2* SNP-edited cells, *PTPN2* expression decreased by 3.2-fold, and proliferation increased by 10.2-fold compared to control. Likewise, expression of *PTPN22* decreased by 2.4-fold and proliferation increased by 8.4-fold in *PTPN22* SNP-edited cells. IFN-γ and TNF-α secretions increased in both edited cell lines. CD25 expression (cell activation) was 80.32% in *PTPN2* SNP-edited cells and 85.82% in *PTPN22* SNP-edited cells compared to 70.48% in unedited Jurkat T-cells. Treatment of *PTPN2* and *PTPN22*-edited cells with a maximum 20 μM spermidine restored *PTPN2/22* expression and cell response including cell proliferation, activation, and cytokines secretion. Most importantly, the effect of spermidine on edited cells restored normal expression and secretion of IFN-γ and TNF-α. The data clearly demonstrated that edited SNPs in *PTPN2* or *PTPN22* were associated with reduced gene expression, which resulted in an increase in cell proliferation and activation and overactive immune response. The data validated our earlier observations in CD and RA clinical samples. Surprisingly, spermidine restored *PTPN2/22* expression in edited Jurkat T-cells and the consequent beneficial effect on cell response and inflammation. The study supports the use of polyamines dietary supplements for management of CD and in RA patients.

## 1. Introduction

Crohn’s Disease (CD) is a chronic inflammatory disease that affects the entire gastrointestinal tract with the most commonly affected segments being the terminal ileum and the colon [1]. Symptoms of CD can include abdominal pain, diarrhea, and weight loss, which can be attributed to ulcer formation in the mucosa [2]. The incidence of CD has been steadily increasing worldwide with greater incidence and prevalence in developed countries and urban areas compared to developing countries and rural areas [3]. The highest prevalence of CD is in Europe, Canada, and the United States where there are approximately 200 to 330 cases per 100,000 people [3,4]. Rheumatoid Arthritis (RA) is classified as an idiopathic autoimmune disease characterized by severe inflammation that leads to joint swelling and destruction of bone and cartilage [5,6]. Like CD, RA incidence has also been increasing with approximately 1% of the world’s population being affected by RA and its prevalence being 0.5–1% in Europe and North America [7]. Of even greater concern is the increased mortality and premature death associated with RA [7]. As prevalence of these disease continue to rise across the globe, the burden of disease also increases making it important that the factors contributing to the pathogenesis of these diseases are identified.

CD and RA result from the interplay of genetic and environmental factors causing an abnormal immune response [1,6,8,9]. Genetic factors contributing to the predisposition for these diseases include single nucleotide polymorphisms (SNPs) affecting alleles of specific genes [5,10,11,12]. Two genes, PTPN2 and PTPN22 (protein tyrosine phosphatase nonreceptor types 2 and 22), important in T-cells encode enzymes that negatively regulate T-cell receptor (TCR) signaling by dephosphorylating and inactivating associated kinases and substrates [8,13,14,15]. This limits the immune response to an antigen and blocks spontaneous activation of T-cells. For this reason, *PTPN2* and *PTPN22* have been identified to be involved in multiple inflammatory and autoimmune disorders [8,13,14,15]. Two SNPS, one in *PTPN2* (rs478582-C) and the other in *PTPN22* (rs2476601-A) have been linked to a potential increased risk of developing CD and RA (Table 1) [5,10]. The *PTPN2* SNP was found in 83% of CD samples and 79% of RA samples compared to about 60% of healthy controls while the *PTPN22* SNP was present in 16% of CD patients and 29% of RA patients in comparison to only 6% of healthy controls [5,10]. *PTPN2:rs478582-C* occurs within intron 3 and results in a nucleotide change from a thymine to a cytosine which is speculated to cause problems during RNA splicing and cause a loss of the protein’s activity upon completion of translation [5,16,17,18,19]. *PTPN22:rs2476601-A* is located in exon 14 and is a base change from a guanine to an adenosine resulting in the arginine amino acid residue at position 620 to be changed to a tryptophan [5,8,10]. This change is within the catalytic portion of the PTPN22 protein and has been theorized to also decrease the protein’s activity [5,16,17,18,19,20]. In clinical CD and RA samples, these SNP mutations led to decreased expression of their corresponding genes, overexpression of the pro-inflammatory cytokine *IFN-γ*, and increased T-cell proliferation indicating their possible role in the pathogenesis of inflammatory autoimmune disorders [5,10]. These findings, however, have not yet been confirmed in vitro.

The most commonly used therapeutic agents for the treatment of inflammatory autoimmune disorders like CD and RA are glucocorticoids, disease-modifying anti-rheumatic drugs (DMARDs), and non-steroid anti-inflammatory drugs (NSAIDs) [22,23]. However, some patients may not respond to these treatments and long-term use of these medications can result in a number of side effects [22,23,24]. For example, continuous use of NSAIDs and glucocorticoids can lead to ulceration, osteoporosis, hypertension, and weight gain among others [22,23,24]. Side effects of DMARDs include gastrointestinal (GI) intolerance, hypersensitivity to the medication, production of antibodies against the medication, and an increased risk of developing opportunistic infections, particularly mycobacterial infections [23,25,26]. There is a need for safer and more effective therapies for patients with inflammatory autoimmune disorders.

Recent research has investigated the effect of treating human intestinal epithelial cells with the *PTPN2:rs1893217-G* SNP that is a risk factor for inflammatory bowel disease (IBD) with different polyamines [27]. They found the natural occurring polyamine, spermidine, was most effective in increasing *PTPN2* expression and activating its phosphatase activity, as well as reducing STAT1 and STAT3 phosphorylation by IFN-γ which indicates its ability to suppress pro-inflammatory signaling cascades [27]. Spermidine treatment also decreased expression of the pro-inflammatory cytokine, *IFN-γ*, and its pro-inflammatory target genes, *ICAM-1* and *NOD2* [27]. These effects were also found to be greater in cells with the *PTPN2:rs1893217-*G SNP compared to wild-type cells [27]. Another study investigated spermidine’s ability and mechanism in treating acute colitis in mice [28]. In the colitis models, spermidine was found to reduce the disease activity index (DAI), reduce colonic inflammation, and increase colonic length [28]. Furthermore, pro-inflammatory cytokine expression, NF-κB (nuclear factor-κB) and MAPK (mitogen-activated protein kinase) phosphorylation, and activation of macrophages and T-cells in the colon decreased with pre- and post-treatment of spermidine [28]. In inflamed colons and a mouse macrophage cell line, there was upregulation of anti-inflammatory M2 macrophage markers and downregulation of pro-inflammatory M1 markers with spermidine treatment [28]. Additionally, LPS/TNF-α-induced inflammation decreased with spermidine treatment in Caco-2 cells [28]. These findings provide strong evidence for the potential use of spermidine as a therapeutic agent for IBD due to its ability to ameliorate colonic inflammation in mice with acute colitis [28].

The present study aimed to confirm the role of the *PTPN2: rs478582-C* and *PTPN22: rs2476601-A* SNPs in the inflammatory pathogenesis associated with Crohn’s Disease and Rheumatoid Arthritis. It also aimed to investigate the anti-inflammatory effects of spermidine for its potential therapeutic use in autoinflammatory immune disease patients with genetic anomalies.

## 2. Materials and Methods

### 2.1. CRISPR-Cas9 Transfection, Stimulation, and Treatment of Jurkat T-Cells

The Jurkat T-cell cell line (ATCC TIB-152) was cultured in RPMI-1640 medium (ATCC 30-2001) with 10% fetal bovine serum (FBS; Sigma Life Science, St. Louis, MO, USA). Cells were maintained in a humidified 5% CO_2_ incubator at 37 °C and grown to confluency in cell culture flasks. A total of 2.0 mL of cell suspension were transferred to 12-well tissue culture plates with 8 × 10^4^ cells per well. Donor DNA duplex was prepared by mixing the donor DNA with its reverse complement (Table 2) in a 1:1 molar ratio in a microcentrifuge tube and then diluting the mixture to a final concentration of 1 pmol/μL with a Tris buffer containing salt—10 mM Tris, 1 mM EDTA, 50 mM NaCl (pH 8.0). The tube was incubated in a heating block at 95 °C for 5 min and the temperature was then gradually reduced until room temperature was reached. In a 2.0 mL microcentrifuge tube (Tube 1), 100 μL of Opti-MEM I Reduced Serum Medium (Thermo Fisher Scientific, Waltham, MA, USA), 5.0 μg of TrueCut Cas9 Protein v2 (Thermo Fisher Scientific, Waltham, MA, USA), 1.0 μg of TrueGuide Synthetic single-guide RNA (sgRNA; see Table 2; Thermo Fisher Scientific, Waltham, MA, USA), 10 μL of Cas9 Plus Reagent (Thermo Fisher Scientific, Waltham, MA, USA), and 1.0 μg of donor DNA duplex (see Table 2; Thermo Fisher Scientific, Waltham, MA, USA) were mixed. In another 2.0 mL microcentrifuge tube (Tube 2), 100 μL of Opti-MEM I Reduced Serum Medium and 6 μL of Lipofectamine CIRSPRMAX Transfection Reagent (Thermo Fisher Scientific, Waltham, MA, USA) were mixed and the solution from Tube 1 was immediately added to Tube 2. After mixing the combined solution, the CRISPR transfection complex was incubated at RT for 5–10 min. Cells were then left untreated or treated with 200 μL of either the *PTPN2* SNP or *PTPN22* SNP CRISPR transfection complexes. A mock transfection control was prepared similarly by using all transfection components except TrueGuide Synthetic sgRNA. Cells were incubated for 3 days before being transferred to a cell culture flask and grown to confluency. Phytohemagglutinin (PHA) is a well-known selective T-cell mitogen and as such was used in this study to stimulate the Jurkat T-cells. A total of 2.0 mL of cell suspension (Wild-type (WT), mock transfection control, *PTPN2* SNP, and *PTPN22* SNP Jurkat T-cells) were transferred to 12-well tissue culture plates with 2 × 10^5^ cells per well and incubated with either RPMI only or 10 μg/mL PHA (Sigma Life Science, St. Louis, MO, USA), and concurrently treated with 0, 10, or 20 μM of spermidine ≥ 99% (GC) (SPD; Sigma-Aldrich, St. Louis, MO, USA) for 72 h, before being subject to further testing.

### 2.2. Confirming Induction of PTPN2/22 SNPs

DNA was isolated from WT, *PTPN2* SNP, or *PTPN22* SNP Jurkat T-cells using the DNeasy Blood and Tissue DNA isolation Kit (QIAGEN, Germantown, MD, USA). Briefly, 2 mL of each cell culture was centrifuged at 2500 rpm for 5 min before resuspending the pellets in 200 μL of PBS. Next, 20 μL of Proteinase K was added to each sample followed by 200 μL of Buffer AL. The tubes were mixed thoroughly by vortexing and then incubated for 10 min at 56 °C. Then, 200 μL of 100% ethanol were added to each sample and mixed via vortexing. Each mixture was then added to a DNeasy Mini spin column placed in a 2 mL collection tube before being centrifuged for 1 min at 8000 rpm. The flow-throughs and collection tubes were discarded while the spin columns were placed in new 2 mL collection tubes and had 500 μL of Buffer AW1 added to them. After centrifuging again at 8000 rpm for 1 min, flow-throughs and collection tubes were discarded and spin columns were added to new 2 mL collection tubes. Next, 500 μL of Buffer AW2 were added to the spin columns before centrifuging for 3 min at 14,000 rpm and discarding the flow-throughs and collection tubes. The spin columns were then placed in a clean 1.5 mL microcentrifuge tube and had 200 μL of Buffer AE added directly to their membranes. After incubating at RT for 1 min, they were centrifuged for 1 min at 8000 rpm to elute DNA. DNA concentrations were measured using NanoDrop (OD at 260 nm). Polymerase Chain Reaction (PCR) was then used to amplify the *PTPN2/22* SNP regions using 25 μL of PCR Master Mix (2X) (Promega, Madison, WI, USA), 15 μL of DNA, and 2 μL of the forward and reverse PCR primers for the *PTPN2* or *PTPN22* SNP regions (Eurofins Scientific, Luxemberg) (Table 2). To conduct the PCR reactions for 30 cycles of: 1 min at 94 °C, 2 min at 54 °C, and 3 min at 72 °C, the MyGene Series Peltier Thermal Cycler was used. PCR samples were then run for 1 h in a 1.5% agarose gel electrophoresis with ethidium bromide to confirm amplification between 300 and 400 bp using 15 μL of PCR product mixed with 5 μL of 6x DNA Loading Dye (Thermo Fisher Scientific, Waltham, MA, USA). The NucleoSpin Gel and PCR Clean-up Kit (Macherey-Nagel, Bethlehem, PA, USA) was used to extract and purify DNA from the agarose gel according to manufacturer’s protocol and DNA was quantified again via NanoDrop (OD at 260 nm) before sending the samples to GENEWIZ for Sanger Sequencing. Sequences were aligned to WT *PTPN2/22* sequences obtained from NCBI (National Center for Biotechnology Information).

### 2.3. Measurement of PTPN2/22 Expression in Treated Jurkat T-Cells

Isolated RNA was reverse-transcribed into cDNA and then used in reverse transcription PCR (RT-qPCR) to determine gene expression levels using primers specific to *GAPDH*, *PTPN2*, and *PTPN22* obtained from Invitrogen (Carlsbad, CA, USA) (Table 3). For RNA isolation, 2.0 mL of treated cells were centrifuged at 2500 rpm for 5 min at 4 °C and the pellets were resuspended in 500 μL of TRIzol reagent (Invitrogen, Carlsbad, CA, USA) and left at RT for 5 min. Then, 125 μL of chloroform was added to each sample and vortexed followed by another 5 min incubation at RT. The samples were centrifuged at 10,000 rpm for 5 min at 4 °C and the upper clear aqueous layers containing RNA were transferred to new 2.0 mL microcentrifuge tubes. Next, each sample was gently mixed with 275 μL of 100% isopropanol, incubated for 5 min at RT, and centrifuged at 14,000 rpm for 20 min at 4 °C. The samples were then placed on ice, the isopropanol was poured off, and 500 μL of 75% ethanol in DEPC-treated water was added. Then, the samples were centrifuged once more at 9500 rpm for 5 min at 4 °C before pouring off the ethanol and leaving the pellets to air-dry for 10 min. Lastly, 15 μL of DEPC-treated water was added to each tube and mixed gently. RNA concentrations were measured using NanoDrop (OD at 260 nm). cDNA was then synthesized from 800 ng of each RNA sample, 4 μL of iScript reverse transcription supermix (BioRad, Hercules, CA, USA), and topped up to a total volume of 20 μL with RNase-free water. MyGene Series Peltier Thermal Cycler was used to conduct the reactions for 5 min at 25 °C, 20 min at 46 °C, and 1 min at 95 °C. cDNA samples were either used immediately afterwards for RT-qPCR analysis or stored at −20 °C. For RT-qPCR, each sample was mixed with 10 μL of Fast SYBR Green Master Mix (Thermo Fisher Scientific, Waltham, MA, USA), 1 μL of forward primer, 1 μL of reverse primer (see Table 3), and 7 μL of DEPC-treated water. Samples were plated in triplicate in a 96-well microamp RT-PCR reaction plate and the 7500 Fast Real-Time PCR System (Applied Biosystems, Foster City, CA, USA) was used to run the experiment. *GAPDH* was used as a control to obtain baseline C_T_ values and gene expression of *PTPN2* and *PTPN22* were measured. Relative gene expression levels were calculated using: (2−∆CT) × 1000, where ∆CT = CT_sample—_CT_GAPDH_.

### 2.4. T-Cell Proliferation Assay of Treated Jurkat T-Cells

In a 96-well culture plate, 100 μL aliquots of 2 × 10^5^/mL of cell suspension (WT, *PTPN2* SNP, or *PTPN22* SNP Jurkat T-cells) were plated per well and incubated with 10 μg/mL PHA and concurrently treated in triplicate with 0, 10, or 20 μM of SPD. The cells were incubated in a humidified 5% CO_2_ incubator at 37 °C for 48 h. The bromodeoxyuridine (BrdU) labeling proliferation ELISA kit was used to perform the T-cell proliferation assay according to manufacturer’s protocol (Abcam, Cambridge, UK). Jurkat T-cells were labeled with 20 μL BrdU and incubated an additional 24 h under the same conditions. Next, 200 μL of fixing solution was added to each well and incubated at RT for 30 min. The plate was washed 3 times with 1× wash buffer before adding 100 μL BrdU monoclonal detector antibody to each well and incubating the plate at RT for 1 h. Then the plate wash was repeated and 100 μL of 1× peroxidase goat anti-mouse IgG conjugate was added to each well and incubated for 30 min at RT. After a final wash step, 100 μL of TMB peroxidase substrate was added to each well and the plate was incubated 30 min in the dark at RT. Finally, 100 μL of stop solution was added to each well and absorbance was read at 450 nm using a spectrophotometric microtiter plate reader. Relative Jurkat T-cell proliferation of samples was compared to WT Jurkat T-cells stimulated with PHA and were determined by calculating the fold change in absorbance readings at 450 nm.

### 2.5. Measurement of IFN-γ and TNF-α Secretion in Treated Jurkat T-Cells

Following 72 h incubation with PHA and spermidine treatment (0, 10, and 20 μM), WT, *PTPN2* SNP, and *PTPN22* SNP Jurkat T-cells were pelleted via centrifugation at 2500 rpm for 5 min at 4 °C. Supernatants were saved and IFN-γ and TNF-α protein secretion levels were determined using the ELLA fully automated ELISA and its Simple Plex analyte cartridges for human IFN-γ 3rd generation and TNF-α 2nd generation (ProteinSimple, San Jose, CA, USA) according to manufacturer’s instructions. All groups were tested in duplicate.

### 2.6. CD25 (IL-2RA) Fluorescent Immunostaining Assay

CD-25 fluorescent immunostaining was performed on WT, *PTPN2* SNP, and *PTPN22* SNP Jurkat T-cells following PHA stimulation and 10 μM of SPD treatment for 72 h. Cells were fixed with 4% paraformaldehyde (PFA) for 30 min. After washing with cold PBS, the cells were blocked with 100 μL of 10% Goat Serum—PBS with Normal Goat Serum (Thermo Fisher Scientific, Waltham, MA, USA)—for 1 h at 25 °C. Next, 100 μL of CD25 Monoclonal Antibody (IL2R.1) (Invitrogen, Carlsbad, CA, USA) diluted 1:10 in 10% Goat Serum was added to the cells and incubated wrapped in aluminum foil in a dark fridge overnight. In the dark, Goat anti-Mouse IgG (H+L) Highly Cross-Adsorbed Secondary Antibody, Alexa Fluor Plus 488 (Invitrogen, Carlsbad, CA, USA) was diluted to 10 μg/mL in PBS, then 100 μL was added to the cells and incubated for 1 h at 25 °C. Cell were washed with cold PBS, and nuclei were then stained using 60 μL VECTASHIELD Antifade Mounting Medium containing 4′,6-diamidino-2-phenylindole (DAPI; Vector Laboratories, Burlingame, CA, USA). Lastly, slides were examined under the AmScope IN480TC-FL-MF603 Fluorescence Microscope, where green staining indicated CD25 expression and blue staining represents nuclei. The NIH Image J 1.39o software (Rockville, MD, USA) was then used to generate merged images and the number of cells expressing CD25 and total number of cells were counted and used to calculate the percentage of CD25-expressing cells.

### 2.7. Statistical Analysis

To analyze data statistics, GraphPad Prism V.7.02 (GraphPad, La Jolla, CA, USA) was used. Significance among experiments was assessed by either Unpaired Two-tailed t test or one-way analysis of variance (ANOVA) followed by Tukey’s multiple comparison test and cross checked with Wilcoxon matched-pairs test for non-parametric tests. Immunostaining data were subject to the z-test. All experiments were performed in triplicates, and data are expressed as average ± SD of the mean and the difference between controls and samples was considered statistically significant at a *p* value < 0.05 and at 95% confidence interval (CI).

## 3. Results

### 3.1. PTPN2/22 SNP Sequencing

Alignment of *PTPN2/22* SNPs sequences region in CRISPR-Cas9-edited Jurkat T-cells with Wild-type *PTPN2/22* sequences showed clear differences between edited cells and the wild-type control. Edited and unedited Jurkat T-cells were sub-cultured and used in this study.

### 3.2. PTPN2/22 SNPs Decrease Gene Expression upon Induction in Jurkat T-Cells Using CRISPR-Cas9

**Figure 1:**
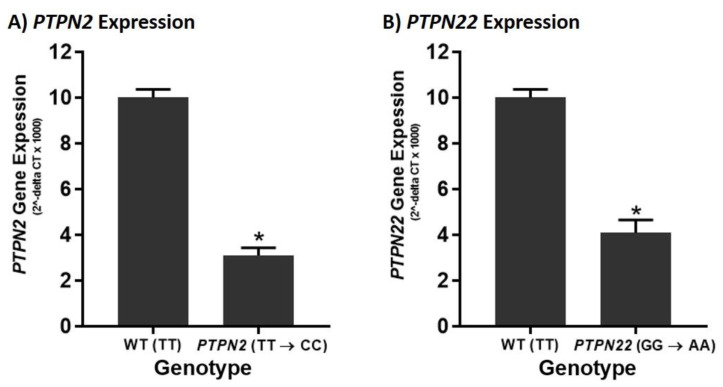
Effect of *PTPN2/22* SNPs on Their Corresponding Gene Expression in CRISPR-Cas9-edited Jurkat T-cells. RT-PCR was used to measure expression of (**A**) *PTPN2* and (**B**) *PTPN22* in WT and CRISPR-Cas9-edited Jurkat T-cells (*PTPN2* SNP and *PTPN22* SNP) (24 h) (*n* = 3). * *p* < 0.05.

**Figure 2:**
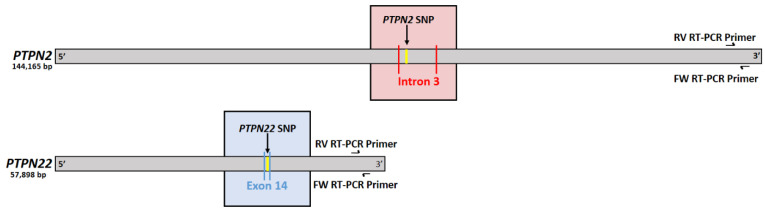
*PTPN2/22* Gene Maps with Relative SNPs and RT-PCR Primer Locations.

The effects of the SNPs, *PTPN2:rs478582-C* and *PTPN22:rs2476601-A*, on their corresponding gene expression was tested in Jurkat T-cells 72 h after transfection with CRISPR-Cas9 and donor DNA (Figure 1). The results indicate that in the presence of the *PTPN2* SNP, expression of *PTPN2* is significantly reduced (Figure 1A). *PTPN2* expression was 3.2-fold lower in *PTPN2* SNP cells compared to WT Jurkat T-cells (3.12 ± 0.332 and 10.0 ± 0.366, respectively). Similar effects were seen for the *PTPN22* SNP, where its presence resulted in a significant reduction in *PTPN22* expression (Figure 1B). *PTPN22* SNP cells had 2.4-fold lower expression of *PTPN22* compared to WT Jurkat T-cells (4.09 ± 0.567 and 10.0 ± 2.35, respectively). Mock transfection control did not have any significant changes in *PTPN2* or *PTPN22* gene expression in comparison to WT jurkat T-cells. Figure 2 depicts the PTPN2/22 gene maps with the relative locations of each SNP and the RT-PCR primers.

### 3.3. PTPN2/22 SNPs Increase T-Cell Proliferation upon PHA Induction in Jurkat T-Cells Using CRISPR-Cas9

**Figure 3:**
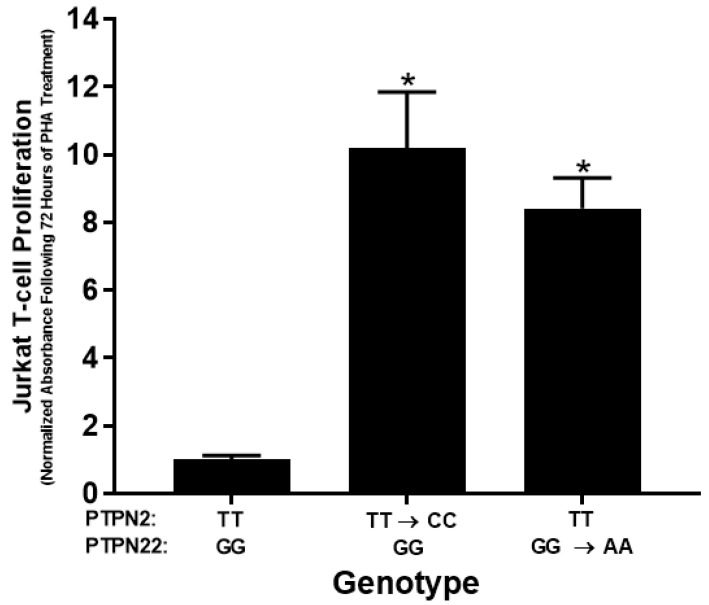
Effect of *PTPN2/22* SNPs on Proliferation in CRISPR-Cas9-edited Jurkat T-cells Stimulated with PHA. BrdU-labeling proliferation ELISA was used to measure proliferation in WT, *PTPN2* SNP, and *PTPN22* SNP Jurkat T-cells stimulated with PHA (10 μg/mL) after 72 h (*n* = 3). * *p* < 0.05.

**Figure 4:**
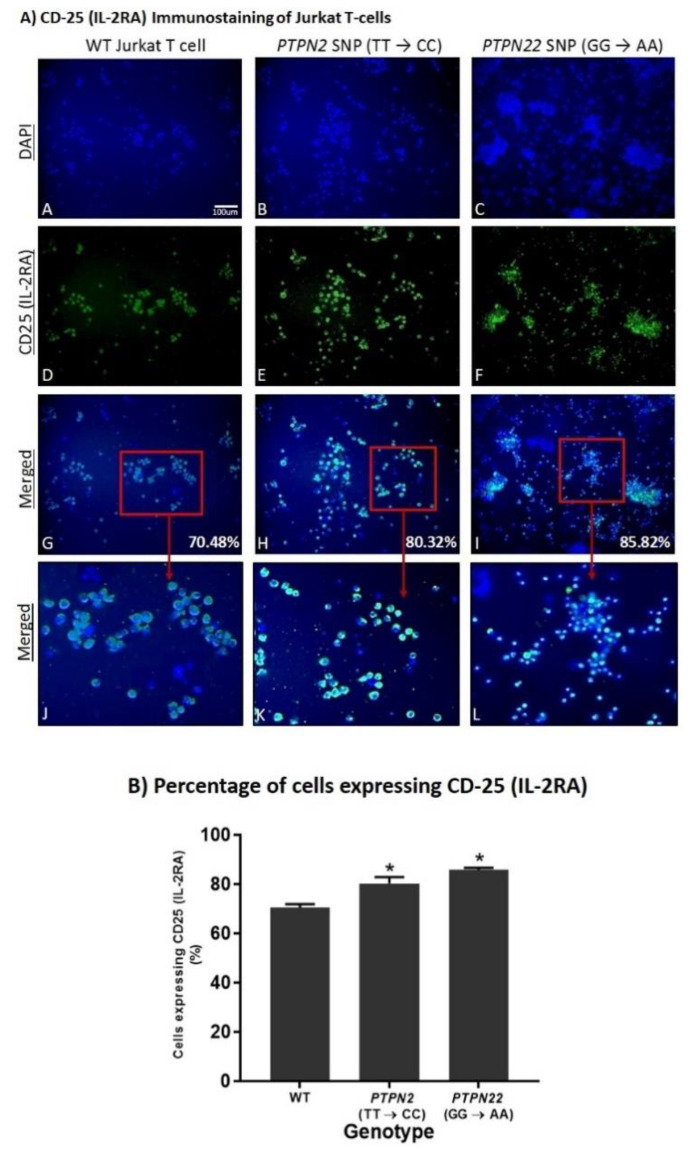
Effect of *PTPN2/22* SNPs on CD25 (IL-2RA) Expression in CRISPR-Cas9-edited Jurkat T-cells Stimulated with PHA. Total nuclei are stained with DAPI in blue (**A**–**C**). CD25 positive cells are stained in green (**D**–**F**), and merged cells are presented with blue and green (**G**–**L**). The histogram (**B**) shows percentage of cells expressing CD25 in each group (WT, *PTPN2* SNP, and *PTPN22* SNP Jurkat T-cells). (*n* = 3). * *p*-value < 0.05.

The effects of the SNPs, *PTPN2:rs478582-C* and *PTPN22:rs2476601-A*, were examined on the proliferation of PHA-stimulated Jurkat T-cells. T-cell proliferation was significantly greater in *PTPN2* and *PTPN22* SNP cells compared to WT Jurkat T-cells. Proliferation levels of Jurkat T-cells with the *PTPN2* SNP increased by 10.2-fold relative to WT cells (10.2 ± 1.64 and 1.00 ± 0.124, respectively) (Figure 3). Likewise, the *PTPN22* SNP increased proliferation levels by 8.4-fold in comparison to WT cells (8.42 ± 0.896 and 1.00 ± 0.124, respectively) (Figure 4). Mock transfection control did not have any significant impact on cell proliferation in comparison to WT jurkat T-cells.

### 3.4. PTPN2/22 SNPs Increase Pro-Inflammatory Cytokine Secretion upon Induction in Jurkat T-Cells Using CRISPR-Cas9

To measure the effects of the *PTPN2:rs478582-C* and *PTPN22:rs2476601-A* SNPs on secretion of the pro-inflammatory cytokines, IFN- γ and TNF-α, by PHA-stimulated Jurkat T-cells, a fully automated ELISA was used. IFN- γ and TNF-α secretion levels were significantly increased in *PTPN2* and *PTPN22* SNP Jurkat T-cells compared to WT cells (Table 4). Specifically, IFN- γ secretion by WT cells were 0.029 ± 0.003 pg/mL compared to *PTPN2* and *PTPN22* SNP cells with 0.060 ± 0.004 pg/mL and 0.054 ± 0.002 pg/mL, respectively (Table 4). Similar trends were seen for TNF-α secretion with WT cells having 0.293 ± 0.042 pg/mL, *PTPN2* SNP cells with 0.507 ± 0.014 pg/mL, and *PTPN22* SNP cells with 0.408 ± 0.039 pg/mL (Table 4).

### 3.5. PTPN2/22 SNPs Increase T-Cell Activation upon Induction in Jurkat T-Cells Using CRISPR-Cas9

Fluorescent immunostaining of CD-25 (IL-2RA) was used on PHA-stimulated Jurkat T-cells to determine the effects of the *PTPN2:rs478582-C* and *PTPN22:rs2476601-A* SNPs on T-cell activation as seen in Figure 4 where blue staining indicates nuclei of cells and green staining represents CD25 expression on the cell surface. The assay showed significantly more cells expressing CD25 (IL-2RA) in the *PTPN2* and *PTPN22* SNP cell groups (80.32 ± 2.6% and 85.82 ± 0.8%, respectively) compared to WT Jurkat T-cells (70.48 ± 1.4%), thus indicating greater T-cell activation levels in those cell groups (Figure 4A,B).

### 3.6. Spermidine Increased PTPN2/22 Expression in CRISPR-Cas9-Edited Jurkat T-Cells with PTPN2/22 SNPs

**Figure 5:**
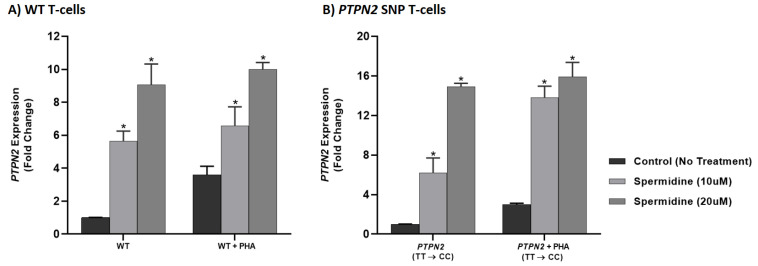
Effect of Spermidine on *PTPN2* Expression in WT and *PTPN2* SNP Jurkat T-cells stimulated with PHA (*n* = 3). RT-PCR was used to measure expression of *PTPN2* in (**A**) WT and (**B**) *PTPN2* SNP T-cells stimulated with PHA (10 μg/mL) and treated with spermidine (0, 10, 20 µM). Significance among experiments was assessed by one-way analysis of variance (ANOVA) followed by Tukey’s multiple comparison test and cross checked with Wilcoxon matched-pairs test for non-parametric tests. * *p* < 0.05 compared to unstimulated or untreated cells for each cell type.

**Figure 6:**
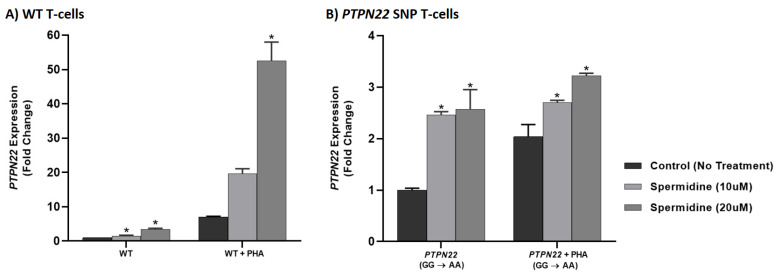
Effect of Spermidine on *PTPN22* Expression in WT and *PTPN22* SNP Jurkat T-cells Stimulated with PHA (*n* = 3). RT-PCR was used to measure expression of *PTPN22* in (**A**) WT and (**B**) *PTPN22* SNP T-cells stimulated with PHA (10 µg/mL) and treated with spermidine (0, 10, 20 µM). Significance among experiments was assessed by one-way analysis of variance (ANOVA) followed by Tukey’s multiple comparison test and cross checked with Wilcoxon matched-pairs test for non-parametric tests. * *p* < 0.05 compared to unstimulated or untreated cells for each cell type.

**Figure 7:**
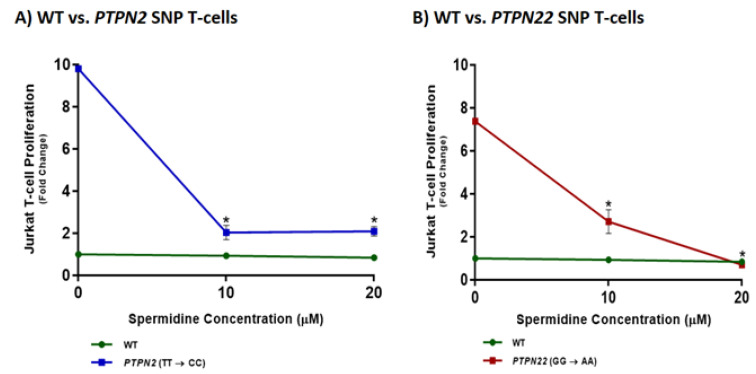
Effect of Spermidine on Proliferation in CRISPR-Cas9-edited Jurkat T-cells Stimulated with PHA. BrdU-labeling proliferation ELISA was used to measure proliferation in (**A**,**B**) WT, (**A**) *PTPN2* SNP, and (**B**) *PTPN22* SNP Jurkat T-cells stimulated with PHA (10 μg/mL) and treated with spermidine (0, 10, 20 μM) (*n* = 3). * *p* < 0.05.

Figures 6 and 7 depict the effect of spermidine treatment (10 and 20 μM) on *PTPN2/22* expression in WT, *PTPN2:rs478582-C,* and *PTPN22:rs2476601-A* SNP Jurkat T-cells as determined via RT-qPCR. In unstimulated WT Jurkat T-cells, 10 μM of spermidine significantly increased *PTPN2* and *PTPN22* expression (5.65 ± 0.604 and 1.53 ± 0.165, respectively) relative to *PTPN2/22* expression in untreated WT cells (1.00 ± 0.018 and 1.00 ± 0.013, respectively) [Figures 6A and 7A]. *PTPN2/22* expression was further increased significantly with 20 μM of spermidine (9.06 ± 1.26 and 3.39 ± 0.352, respectively) (Figures 6A and 7A). Relative to *PTPN2* expression in PHA-stimulated WT Jurkat T-cells not treated with spermidine (3.61 ± 0.512), *PTPN2* expression was significantly increased with 10 and 20 μM of spermidine (6.56 ± 1.16 and 9.98 ± 0.430, respectively) (Figure 5A). Although PTPN22 expression in PHA-stimulated WT Jurkat T-cells increased with 10 and 20 μM of spermidine (19.6 ± 1.49 and 52.6 ± 5.38, respectively), the increase was only significant with 20 μM compared to PHA-stimulated WT Jurkat T-cells with no spermidine (7.08 ± 0.162) [Figure 6A]. Spermidine significantly increased *PTPN2* expression in unstimulated and PHA-stimulated *PTPN2* SNP cells in a dose-dependent manner (Figure 5B). In comparison to *PTPN2* expression in unstimulated *PTPN2* SNP Jurkat T-cells not treated with spermidine (1.00 ± 0.020), those treated with 10 and 20 μM of spermidine had expression increase to 6.19 ± 1.51 and 14.9 ± 0.354, respectively (Figure 5B). The same was seen for *PTPN2* expression in PHA-stimulated *PTPN2* SNP cells treated with 10 and 20 μM of spermidine (13.8 ± 1.16 and 15.9 ± 1.46, respectively) when compared to PHA-stimulated *PTPN2* SNP cells alone (3.01 ± 0.111) (Figure 5B). Spermidine treatment also significantly increased *PTPN22* expression in unstimulated and PHA-stimulated *PTPN22* SNP Jurkat T-cells in a dose-dependent manner (Figure 6B). Relative to *PTPN22* expression in unstimulated *PTPN22* SNP cells with no spermidine treatment (1.00 ± 0.038), 10 and 20 μM of spermidine increased expression to 2.46 ± 0.064 and 2.57 ± 0.383, respectively (Figure 6B). Similarly, *PTPN22* expression increased to 2.70 ± 0.044 with 10 μM of spermidine and 3.22 ± 0.051 with 20 μM of spermidine in *PTPN22* SNP cells compared to untreated PHA-stimulated *PTPN22* SNP cells (2.04 ± 0.233) (Figure 6B).

### 3.7. Spermidine Decreases T-Cell Proliferation in CRISPR-Cas9-Edited Jurkat T-Cells with PTPN2/22 SNPs

The effect of spermidine (10 and 20 μM) was examined on T-cell proliferation in PHA-stimulated WT, *PTPN2:rs478582-C,* and *PTPN22:rs2476601-A* SNP Jurkat T-cells (Figure 7). Spermidine did not have any effect on WT Jurkat T-cell proliferation levels at 10 nor 20 μM (2.04 ± 0.344 and 0.847 ± 0.026, respectively) relative to untreated WT Jurkat T-cells (1.00 ± 0.124). In comparison to proliferation levels of untreated *PTPN2* SNP cells (10.2 ± 1.64), those treated with 10 and 20 μM of spermidine had a significant reduction in proliferation (2.04 ± 0.344 and 2.09 ± 0.231, respectively) (Figure 7A). A similar trend was seen for *PTPN22* SNP cells, where 10 and 20 μM of spermidine significantly reduced proliferation levels relative to untreated *PTPN22* SNP cells (2.71 ± 0.554, 0.703 ± 0.017, and 8.42 ± 0.896, respectively) (Figure 7B).

### 3.8. Spermidine Decreases Pro-Inflammatory Cytokine Secretion in CRISPR-Cas9-Edited Jurkat T-Cells with PTPN2/22 SNPs

In Table 5, a fully automated ELISA was again used to evaluate pro-inflammatory cytokine secretion levels in PHA-stimulated WT, *PTPN2:rs478582-C*, and *PTPN22:rs2476601-A* SNP Jurkat T-cells treated with spermidine (10 and 20 μM). IFN-γ and TNF-α levels in PHA-stimulated WT Jurkat T-cells with no spermidine treatment were 0.283 ± 0.026 pg/mL and 0.785 ± 0.012 pg/mL, respectively. Relative to these untreated controls, 10 and 20 μM of spermidine showed a significant reduction in IFN-γ secretion with 0.124 ± 0.005 pg/mL and 0.172 ± 0.010 pg/mL, respectively. Similarly, TNF-α secretion levels were significantly reduced in response to 10 and 20 μM of spermidine (0.359 ± 0.012 pg/mL and 0.353 ± 0.022 pg/mL, respectively). PHA-stimulated *PTPN2* SNP Jurkat T-cells with no spermidine treatment IFN-γ and TNF-α secretion levels of 0.359 ± 0.020 pg/mL and 4.87 ± 0.292 pg/mL, respectively. Relative to these untreated controls, 10 and 20 μM of spermidine significantly reduced IFN-γ levels (0.115 ± 0.010 pg/mL and 0.164 ± 0.006 pg/mL, respectively). The same was seen for TNF-α levels with spermidine treatment of 10 μM (0.469 ± 0.015 pg/mL) and 20 μM (0.484 ± 0.016 pg/mL). Untreated *PTPN22* SNP Jurkat T-cells stimulated with PHA had IFN- γ secretion of 0.237 ± 0.009 pg/mL and TNF-α secretion of 10.8 ± 0.318 pg/mL. In comparison to these untreated controls, 10 μM of spermidine significantly reduced IFN- γ secretion (0.108 ± 0.010 pg/mL) as well as TNF- α secretion (0.609 ± 0.021 pg/mL). The same was seen for 20 μM of spermidine where IFN-γ secretion was 0.130 ± 0.010 and TNF-α secretion was 0.408 ± 0.039 pg/mL. 

## 4. Spermidine Decreases T-Cell Activation in CRISPR-Cas9-Edited Jurkat T-Cells with PTPN2/22 SNPs

**Figure 8:**
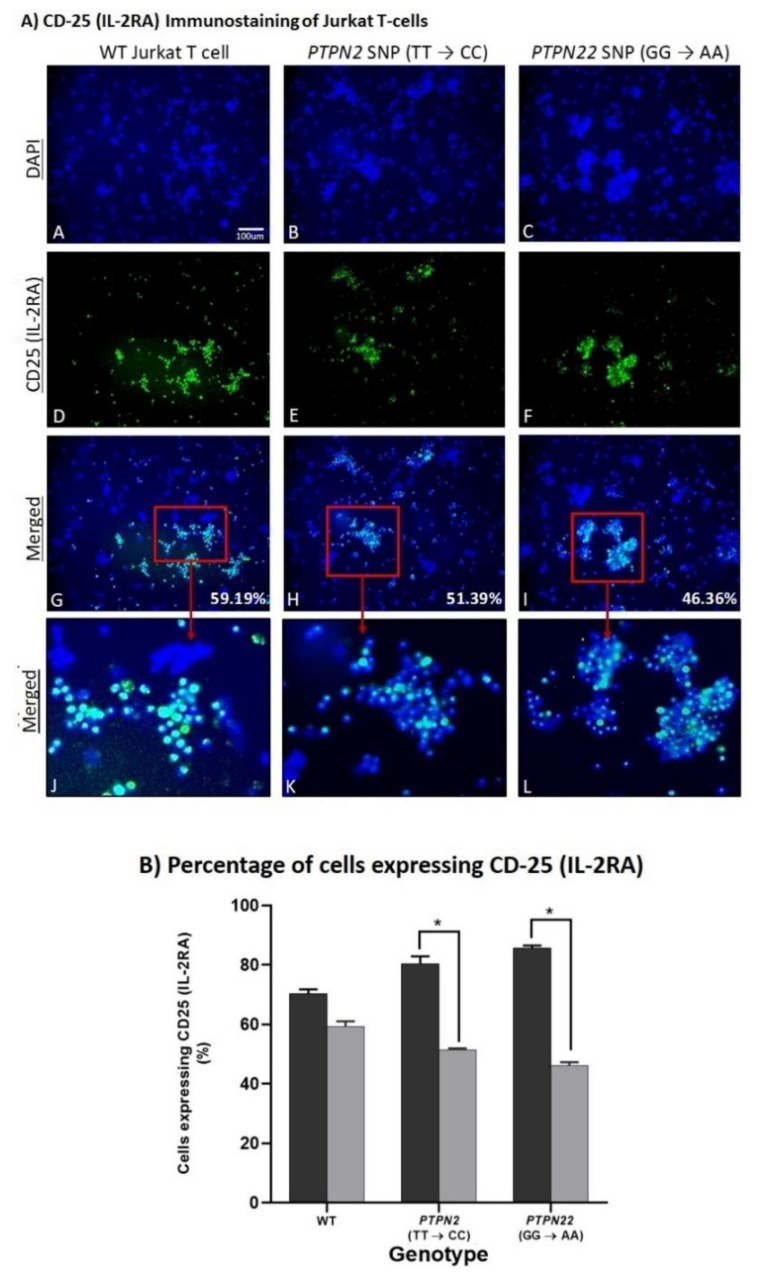
Effect of Spermidine on CD25 (IL-2RA) Expression in CRISPR-Cas9-edited Jurkat T-cells Stimulated with PHA. Total nuclei are stained with DAPI in blue (**A**–**C**). CD25 positive cells are stained in green (**D**–**F**), and merged cells are presented with blue and green (**G**–**L**). The histogram (**B**) shows percentage of cells expressing CD25 in untreated and spermidine treated (0, 10, 20 μM) cells (WT, *PTPN2* SNP, and *PTPN22* SNP Jurkat T-cells). (*n* = 3). * *p*-value < 0.05.

Fluorescent immunostaining of CD-25 (IL-2RA) was used again to determine the effect of 10 μM of spermidine on activation of PHA-stimulated WT, *PTPN2:rs478582-C,* and *PTPN22:rs2476601-A* SNP Jurkat T-cells as seen in Figure 8. The results showed a significant reduction in the number of cells expressing CD25 (IL-2RA) in the *PTPN2* and *PTPN22* SNP cell groups upon spermidine treatment which indicates decreased Jurkat T-cell activation levels (Figure 8). In comparison to 80.32 ± 2.6% of untreated *PTPN2* SNP cells expressing CD25, only 51.39 ± 0.6% of *PTPN2* SNP cells treated with 10 μM of spermidine expressed CD25. (Figure 8B). Similarly, 46.36 ± 1.00% of spermidine treated *PTPN22* SNP cells expressed CD25 as opposed to 85.82 ± 0.8% of untreated PTPN22 SNP cells (Figure 8B). There was a decrease in CD-25 expressing cells in WT Jurkat T-cells treated with spermidine (59.19 ± 1.9%) compared to untreated WT Jurkat T-cells (70.5 ± 1.4%), however, the difference was not significant (Figure 7 and Figure 8).

## 5. Discussion

Inflammatory disorders such as CD and RA result from a combination of both genetic predisposition and an environmental trigger [1,6,8,9]. These genetic SNPs cause an increased immune response, which is then made worse by environmental triggers [5,10]. Of added importance is the need for cheaper and safer therapeutic agents, with fewer side effects, that are effective in patients with genetic polymorphisms. It is crucial that we understand the etiology and contributing factors to the pathogenesis of these disorders so improved diagnostics and therapies can be identified and developed.

Our lab previously identified the *PTPN2* SNP (rs478582-C) and the *PTPN22* SNP (rs2476601-A) as being significant in clinical samples of CD and RA [5,10]. Patient samples with these SNPs were found to have increased inflammatory processes compared to normal, WT, patient samples [5,10]. This intrigued us to investigate the inflammatory effects of these *PTPN2/22* SNPs in vitro to confirm and validate our previous findings in pure Jurkat T-cell cultures without confounding variables that may influence the observed response as seen in clinical samples.

This study confirmed the pro-inflammatory effects of the *PTPN2/22* SNPs in human T lymphocytes and evaluated the anti-inflammatory effects of spermidine in normal cells and those with genetic anomalies. This was achieved by using CRISPR-Cas9 to induce the *PTPN2/22* SNPs in the genomic DNA of Jurkat T-cells and measuring their effect on *PTPN2/22* gene expression, T-cell proliferation, pro-inflammatory cytokine secretion, and T-cell activation, which are all reportedly effected in CD and RA patients [5,10]. While further verification of the SNP region sequencing is pending, we presume to have mixed cell cultures of WT and edited cells based on all the compiled results indicating increased inflammatory responses in SNP cell groups. Thus, our findings of inflammation induced by the *PTPN2/22* SNPs may be of smaller magnitude compared to a pure culture of *PTPN2/22* SNP cells.

There has been extensive debate in the literature on the effect of *PTPN2/22* SNPs on their corresponding gene expression and function [8,29,30,31,32]. Our results showed that presence of the *PTPN2/22* SNPs resulted in a significant decrease in expression of their corresponding genes; *PTPN2* SNP cells had lower *PTPN2* expression and *PTPN22* SNP cells had lower *PTPN22* expression compared to WT cells. This reduction could lead to loss of the negative regulatory function of *PTPN2* and *PTPN22*. Furthermore, this decrease in *PTPN2/22* expression in edited Jurkat T-cells was at a markedly greater reduction than our lab’s previous findings using clinical blood samples from CD and RA patients. High T-cell proliferation levels also contribute to the progressive inflammation associated with CD and RA [5,10,33,34,35]. Stimulation of edited Jurkat T-cells with PHA resulted in a significantly greater proliferation response than in WT Jurkat T-cells. *PTPN2* SNP cells had a 10.2-fold increase while *PTPN22* SNP cells had an 8.4-fold increase. This increase in proliferation was even greater than previous findings using clinical CD and RA samples where there was only a 2.2-fold and 1.2-fold increase, respectively [5,10,36]. CD and RA are also characterized by an increase in the pro-inflammatory cytokines IFN-γ and TNF-α [5,10,37]. In agreement with this, this study showed a significant increase in secretion of both IFN-γ and TNF-α in edited Jurkat T-cells in comparison to WT Jurkat T-cells. Lastly, CD and RA also consist of hyperactive T-cells [5,10,33,34,35]. The most prominent marker of T-cell activation is CD25 or IL-2RA which is the alpha chain of the trimeric IL-2 receptor on the surface of many peripheral blood lymphocyte subsets [38]. Stimulation of the TCR complex by IL-2 or other cytokines released by monocytes or macrophages activates T-cells leading to upregulation of CD25 expression [38]. CD25 expression then triggers a signaling cascade that leads to proliferation and survival of activated T-cells [38]. Our findings show a significant increase in the number of Jurkat T-cells expressing CD-25 (IL-2RA) on the cell surface in *PTPN2* and *PTPN22* SNP cell groups, thus indicating hyperactivity.

Our findings thus far confirmed that presence of *PTPN2/22* SNPs leads to a lack of negative feedback regulation resulting in hyperactive Jurkat T-cells that have increased levels of proliferation and pro-inflammatory cytokine secretion. Additionally, the effects seen in the aforementioned results were at a higher magnitude than those found in our lab previously using clinical samples. This could be a result of using pure cell cultures that only vary in the induced genetic SNPs rather than buffy coats collected from blood samples, which contained multiple T-cell subpopulations and have a number of other variables influencing the response such as varying diets or medications among patients. Further research can investigate the effects of *PTPN2/22* SNPs on levels of anti-inflammatory cytokines like IL-6 and confirm their role in promoting inflammation due to a loss of negative regulatory function of *PTPN2/22* by inducing the SNPs in mice models.

A growing field of research is evaluating the anti-inflammatory properties of polyamines. These naturally occurring compounds are central to many pathways involved in cell proliferation, growth, and death [39,40]. Supplementation of polyamines has been proven to promote longevity in a number of organisms including yeast, flies, worms, mice, and human peripheral blood mononuclear cells (PBMCs) [39,40,41,42]. Polyamines also have a central role in reestablishing the intestinal mucosa via proliferation and differentiation [39,43]. In inflammatory disease models, spermidine was shown to have significant anti-inflammatory effects making it a promising candidate for use as a therapeutic agent. It inhibited skin inflammation and macrophage activation in mice, blocked NF-kB and MAPK signaling pathways, reduced pro-inflammatory genes and cytokines expression, modulated M1/M2 macrophage markers, [27,28,44,45].

Our results showed spermidine treatment rescued *PTPN2/22* function in Jurkat T-cells with *PTPN2/22* SNPs. First, spermidine significantly increased *PTPN2/22* expression in all cell types (WT, *PTPN2* SNP, and *PTPN22* SNP) and this increase was in a dose-dependent manner for edited Jurkat T-cells where 20 μM of spermidine resulted in a greater increase than 10 μM. While WT Jurkat T-cell proliferation levels remained relatively constant with spermidine treatment, *PTPN2* and *PTPN22* SNP Jurkat T-cells displayed a significant reduction in proliferation levels when treated with spermidine. In addition, spermidine treatment also significantly reduced secretion of IFN-γ and TNF-α across all cell types. Finally, although spermidine treatment did not significantly reduce the number of WT cells expressing CD25, there was a significant reduction in *PTPN2* and *PTPN22* SNP Jurkat T-cells, indicating a substantial decrease in T-cell activation levels. It is worth mentioning that *PTPN2* in particular is upregulated in response to oxidative stress, and polyamines serve as a substrate for oxidation reactions that lead to hydrogen peroxide (H_2_O_2_) production [46,47]. Therefore, supplementing cell media with antioxidants such as aminoguanidine hydrochloride should elucidate whether spermidine has a direct effect on altering gene expression or its extracellular oxidation is responsible for the observed effects due to H_2_O_2_ production.

Overall, the above results show spermidine reverses the inflammation induced by the disease associated *PTPN2/22* SNPs, providing strong evidence for the consideration of spermidine as a more effective therapeutic agent to treat inflammatory disorders such as CD and RA. However, more research is needed to understand the exact mechanism by which polyamines influence these responses as well as the safety and tolerability of long-term polyamine supplementation. We believe that expression of *PTPN2/22* will be the same and even more significant in a truly pure cell culture, where 100% of T-cells carry both SNPs. Likewise, the effect of spermidine on the restoration of PTPN2/22 expression and function will lead to a similar outcome in pure cell culture samples.

## Figures and Tables

**Table 1:** List of SNPs examined in this study.

Gene	RefSNP	Mutation	Location	Mean Allele Frequency	Mutation Phenotype	Reference
*PTPN2*	rs478582	T → C	Intron 3	C = 0.3158	High susceptibility to RA, TID, MS, and Celiac disease	[16]
*PTPN22*	rs2476601	G → A	R620W	A = 0.0588	High susceptibility to CD, RA, TID, MS, SLE, and Celiac disease	[20]

NCBI was used to obtain gene mutation, location, and mean allele frequency information [21]. R = arginine, W = tryptophan, AA = amino acid, CD = Crohn’s Disease, RA = Rheumatoid Arthritis, TID = type I diabetes, MS = multiple sclerosis, SLE = systemic lupus erythematosus.

**Table 2:** Synthetic single-guide RNA and donor DNA sequences.

Gene	Component	Sequence (5′ → 3′)
*PTPN2*	sgRNA	AUUAUACUACGUCAAUUCAC +scaffold
	Donor DNA	CTACCTCAAGTAAAAAATGCATTTTAGTTTCCTGTGAATT GACGCAGTATAATAGCTCATAGTTACATTAATCTGCATAT (80 bases)
	Donor DNA reverse complement	ATATGCAGATTAATGTAACTATGAGCTATTATACTGCGTC AATTCACAGGAAACTAAAATGCATTTTTTACTTGAGGTAG (80 bases)
	Forward PCR Primer	CAGGGCTGTCTTTCCCCCTA (20 bases)
	Reverse PCR Primer	GCAAAGTGTCCACCTTTGAT (20 bases)
*PTPN22*	sgRNA	AAUGAUUCAGGUGUCCGUAC +scaffold
	Donor DNA	AGCTTCCTCAACCACAATAAATGATTCAGGTGTCC TACAGGAAGTGGAGGGGGGATTTCATCATCTATCC (70 bases)
	Donor DNA reverse complement	GGATAGATGATGAAATCCCCCCTCCACTTCCTGTA GGACACCTGAATCATTTATTGTGGTTGAGGAAGCT (70 bases)
	Forward PCR Primer	CGCCCAGCCCTACTTTTGAG (20 bases)
	Reverse PCR Primer	CCATGCCCATCCCACACTTT (20 bases)

**Table 3:** RT-qPCR primer sequences for tested genes.

Gene	Forward Primer Sequence (5′ → 3′)	Reverse Primer Sequence (5′ → 3′)
*GAPDH*	5′-CTTTTGCAGACCACAGTCCATG-3′ (22 bases)	5′-TTTTCTAGACGGCAGGTCAGG-3′ (21 bases)
*PTPN2*	5′-CTAGAGGGTTAGCGAGCCTCA-3′ (21 bases)	5′- TCATGTGGGAATGATTTTTGGTCAC-3′ (25 bases)
*PTPN22*	5′-TAGTTTTATTTGCAGGTGTACTTGCAG-3′ (27 bases)	5′-TGGTCAAGATGCTGCCTAACATT-3′ (23 bases)

**Table 4:** Effect of *PTPN2/22* SNPs on IFN-γ and TNF-α Secretion in CRISPR-Cas9-edited Jurkat T-cells Stimulated with PHA.

Cell Group	[IFN-γ] ± SD (pg/mL)	[TNF-α] ± SD (pg/mL)
WT	0.029 ± 0.003	0.293 ± 0.042
*PTPN2* (TT → CC)	0.060 ± 0.004 *	0.507 ± 0.014 *
*PTPN22* (GG → AA)	0.054 ± 0.002 *	0.408 ± 0.039 *

(*n* = 2) * *p* < 0.05.

**Table 5:** Effect of Spermidine on IFN-γ and TNF-α Secretion in CRISPR-Cas9-edited Jurkat T-cells Stimulated with PHA.

Treatment	Spermidine Concentration (μM)	IFN-γ ± SD (pg/mL)	TNF-α ± SD (pg/mL)
WT	-	0.029 ± 0.003	0.293 ± 0.042
WT+PHA	-	0.283 ± 0.026	0.785 ± 0.012
	10	0.124 ± 0.005 *	0.359 ± 0.012 *
	20	0.172 ± 0.010 *	0.353 ± 0.022 *
*PTPN2*	-	0.060 ± 0.004	0.408 ± 0.039
*PTPN2*+PHA	-	0.359 ± 0.020	4.87 ± 0.292
	10	0.115 ± 0.010 *	0.469 ± 0.015 *
	20	0.164 ± 0.006 *	0.484 ± 0.016 *
*PTPN22*	-	0.054 ± 0.002	0.507 ± 0.014
*PTPN22*+PHA	-	0.237 ± 0.009	10.8 ± 0.318
	10	0.108 ± 0.010 *	0.609 ± 0.021 *
	20	0.130 ± 0.010 *	0.408 ± 0.039 *

(*n* = 3) * *p* < 0.05.

## Data Availability

All experiments were performed in accordance with relevant guidelines and regulations. Raw data is available upon request.
